# Sea Urchin Pigment Ethylspinazarin (U-573): A Novel P2X7 Receptor Antagonist with Neuroprotective and Antiparkinsonian Effects

**DOI:** 10.3390/ijms26178639

**Published:** 2025-09-05

**Authors:** Evgeny Pislyagin, Sergey Kozlovskiy, Irina Agafonova, Ekaterina Menchinskaya, Ekaterina Chingizova, Tatiana Gorpenchenko, Anatolii Mirochnik, Elena Fedorenko, Yuri Sabutski, Sergei Polonik, Dmitry Aminin

**Affiliations:** 1 G.B. Elyakov Pacific Institute of Bioorganic Chemistry, Far-Eastern Branch of the Russian Academy of Science, 690022 Vladivostok, Russia; agafonova@piboc.dvo.ru (I.A.); ekaterinamenchinskaya@gmail.com (E.M.); martyyas@mail.ru (E.C.); alixar2006@gmail.com (Y.S.); sergpol007@mail.ru (S.P.); daminin@piboc.dvo.ru (D.A.); 2 Federal Scientific Center of East Asia Terrestrial Biodiversity, Far Eastern Branch of the Russian Academy of Sciences, 690022 Vladivostok, Russia; gorpenchenko@biosoil.ru; 3 Institute of Chemistry, Far Eastern Branch of the Russian Academy of Sciences, 690022 Vladivostok, Russia; mirochnik@ich.dvo.ru (A.M.); gev@ich.dvo.ru (E.F.); 4 Department of Biomedical Science and Environmental Biology, Kaohsiung Medical University, Kaohsiung 80708, Taiwan

**Keywords:** 1,4-naphthoquinones, Parkinson’s disease, P2X7R, neuronal cells, neuroprotection

## Abstract

The ability of the quinonoid sea urchin pigment ethylspinazarin (U-573) to protect mouse Neuro-2a neuronal cells from the neurotoxic effect of one of the Parkinson’s disease inducers, MPP+, was studied. This compound blocked Ca^2+^ influx and inhibited macropore formation through the P2X7 receptor induced by high concentrations of ATP. Ethylspinazarin at a concentration of 10 μM increased the viability of neuronal cells treated with the neurotoxin by approximately 15% and reduced the level of NO and ROS to control values. Further, U-573 prevented the MPP+-induced formation of amyloid-like protein aggregates in neuronal cells by approximately 50%. This compound at a dosage of 1 mg/kg exerted an anti-inflammatory effect in a mouse model of inflammation, reducing ATP-induced paw edema to values of intact animals. Moreover, the potential of ethylspinazarin in providing an antiparkinsonian effect was shown using a mouse model of MPTP-induced Parkinson’s disease. It is likely that the antiparkinsonian activity in in vivo experiments may be mediated by the ability of U-573 to cross the blood–brain barrier. Finally, we found that U-573 effectively inhibits the functioning of ATP-dependent purinergic P2X7 receptors in neuronal cells. This property may be of key importance in the manifestation of the antiparkinsonian activity of this 1,4-naphthoquinone.

## 1. Introduction

Parkinson’s disease is a progressive neurodegenerative disorder that causes movement disorders such as tremor, rigidity, and slowness of movement. The severity of symptoms and the speed at which they develop can vary greatly from patient to patient, significantly reducing quality of life if not treated promptly. Existing drugs for PD treatment do not eliminate the cause of the disease but only slow down its development and partially improve the quality of patient life. All these drugs have undesirable side effects, which significantly limits their use. Natural compounds, including herbal remedies and metabolites of marine origin, often have multitarget neuroprotective properties. In addition to antioxidant and anti-inflammatory activity, they are able to restore the functions of lysosomes and mitochondria, inhibit iron accumulation, prevent protein folding disorders, and protect a number of other key molecular mechanisms, the dysfunction of which leads to PD. These compounds are often safe, can increase bioavailability, and mitigate the side effects of existing drugs. All this opens up a broad prospect for the development of new antiparkinsonian agents based on natural products for the treatment of Parkinson’s disease [1,2,3].

The P2X family of purinergic receptors are ion channels activated by exogenous or endogenous adenosine triphosphate (ATP). These receptors are hetero- or homomers that form transport channels that allow cations such as sodium, potassium, and calcium to enter the cell upon activation. P2X7 receptor (P2X7R) is a member of the P2X family that has unique structural and functional characteristics, including lower ATP sensitivity than other members [4].

P2X7R is a single-polypeptide transmembrane protein consisting of approximately 595 amino acids. It forms a homotrimer, an ion channel comprising three identical subunits, each containing two transmembrane domains, spontaneous and structural tethers, and cytoplasmic terminals. A unique feature of P2X7R is a long cytoplasmic terminal of approximately 200 amino acids, which is involved in the regulation of function and interaction with intra- and extracellular messengers [5].

P2X7R also non-selectively passes Na^+^, K^+^, and Ca^2+^ ions, but when exposed to high extracellular concentrations of ATP, it is capable of forming a macropore that allows the passage of positively charged molecules weighing up to 900 Da [6].

P2X7R plays an important role in the activation of macrophages and other immune cells, which affects inflammatory processes and may be involved in the pathogenesis of various diseases [7]. Long-term activation of P2X7R can initiate apoptosis (programmed cell death) or pyroptosis (inflammatory cell death) [8]. These receptors are widely expressed in the brain (especially in microglial cells) and is important in the induction of neuroinflammation and neuronal health [9]. Research on the P2X7R is actively conducted in the context of various diseases, including autoimmune diseases, neurological disorders, metabolic disorders and cancer [10,11,12,13].

P2X7R is one of the key mediators of inflammation and immune response [7]. Various brain injuries caused by chronic neurodegenerative diseases lead to a massive release of ATP through the disrupted cell membranes of microglial and neuronal cells [14]. Stimulation of P2X7R by ATP results in the production of pro-inflammatory cytokines, chemokines, proteases and leads to oxidative stress in cells [15]. Its activation may also be an aggravating factor leading to further neurodegeneration and excitotoxicity in Parkinson’s disease (PD), and is associated with abnormal deposition of alpha-synuclein (α-Syn) [16].

In particular, the activation of P2X7R leads to the initiation of the assembly of the pro-inflammatory NLRP3 inflammasome, which is responsible for the activation of caspases 1 and 3 and the production of pro-inflammatory cytokines IL-1β and IL-18 [17]. Patients with PD have been reported to exhibit systemic hyperexpression of the P2X7R/NLRP3 platform, which is reduced by treatment [18]. Therefore, bioavailable and selective P2X7R antagonists that are able to pass through the blood–brain barrier (BBB) may be effective for the treatment of the consequences of neurodegenerative processes.

1,4-Naphthoquinones are a class of compounds with a broad spectrum of biological activity, which has great potential for the synthesis of derivatives with new types of activity [19]. Previously, a number of studies, including ours, have shown that 1,4-naphthoquinone derivatives can act as promising P2X7R antagonists. Thus, it was demonstrated that the synthesized tetracyclic 1,4-naphthoquinone thioglucoside conjugate U-556, acyclic thioglucoside U-548 and its tetracyclic derivative U-286 can inhibit P2X7R functions in Neuro-2a mouse neuroblastoma cells and RAW 264.7 macrophage cells. These compounds also had antioxidant properties, reduced the production of anti-inflammatory cytokines, and showed anti-inflammatory and analgesic activity in vivo [20,21,22,23]. The other 1,4-naphthoquinone derivatives—2-hydroxy-3-iodo-1,4-NQs (AN-03), 2-hydroxy-3-phenylnaphthalene-1,4-dione (AN-04) and A740003—inhibited P2X7R-induced dye uptake, release IL-1β, and have an anti-inflammatory effect on carrageenan-induced paw edema in vivo [24]. The activity of P2X7R was also significantly suppressed by 2-amino-3-aryl-1,4-naphthoquinones [25].

It has been previously shown that compounds U-443 (acetylated 1,4-naphthoquinone-*O*-glucoside) and the natural pigment of the sea urchin *Scaphechinus mirabilis*. U-573 (6-ethyl-2,3,5,8-tetrahydroxy-1,4-naphthoquinone (ethylspinazarin)) are able to protect mouse neuroblastoma Neuro-2a cells from the toxic effects of rotenone in vitro [26]. This compound also reduced the levels of reactive oxygen species (ROS) and nitric oxide (NO) in neuronal cells and had antioxidant properties. U-443 and U-573 also restored mitochondrial cell functions (mitochondrial potential) under the influence of neurotoxins. This compound significantly reduced the production of pro-inflammatory cytokines TNF and IL-1β, and it inhibited the activity of the pro-inflammatory enzyme cyclooxygenase-2 (COX-2). In vivo studies have shown that the treatment with U-443 and U-573 significantly improves behavioral responses of animals at the early stage of rotenone-induced Parkinson’s disease [26,27].

**Figure 1:**
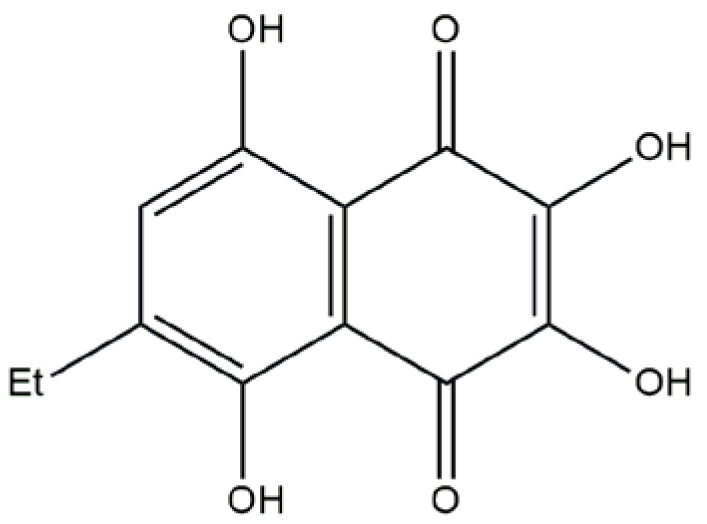
Structure of ethylspinazarin (U-573).

Compound U-573 (Figure 1) has been recognized as one of the most effective 1,4-naphthoquinones with neuroprotective properties. However, nothing is known about the ability of U-573 to block P2X7R, and it is unclear whether this blockade is associated with the anti-inflammatory and antiparkinsonian activity of this 1,4-naphthoquinone derivative. Therefore, in the present work, we studied the ability of U-573 to protect mouse neuronal Neuro-2a cells from the toxic effect of ATP and one of the neurotoxin and PD inducers, MPP+ (1-methyl-4-phenylpyridinium). The capacity of compound U-573 to block calcium ion influx via P2X7R and inhibit ATP-induced macropore formation has been demonstrated. Furthermore, compound U-573’s effectiveness in reducing inflammation as well as the formation of ROS and NO in neuronal cells exposed to ATP and MPP+ was evaluated. Moreover, the involvement of MPP+ in the formation of amyloid-like protein aggregates in neuronal cells, as well as the ability of U-573 to prevent this aggregation, was assessed using the selective fluorescent probe CRANAD-2. Additionally, the compound’s potential to provide anti-inflammatory and antiparkinsonian effects was investigated in vivo using a mouse model of ATP-induced inflammation and MPTP-induced Parkinson’s disease. In conclusion, we studied the ability of U-573 to pass through the BBB.

## 2. Results

### 2.1. U-573 Protects Neuro-2a Cells Against Toxic Concentrations of MPP+ and ATP

**Figure 2:**
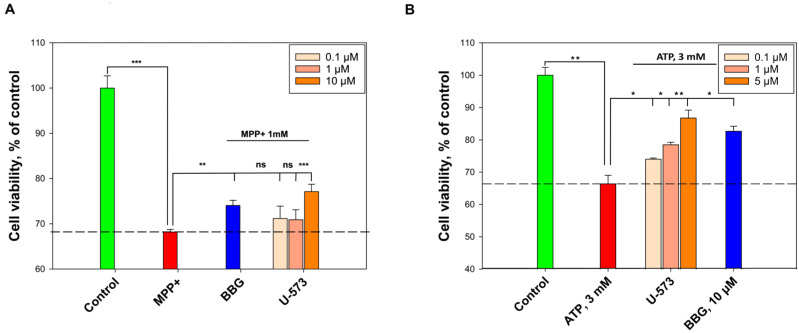
U-573 increases the viability of Neuro-2a under toxic treatment. The effect of U-573 on the viability of Neuro-2a cells under the action of MPP+ (1 mM) (**A**) and ATP (3 mM) (**B**). Data are presented as mean ± SE (*n* = 6). * *p* < 0.05, ** *p* < 0.01, *** *p* < 0.001 compared to MPP+ or ATP; ns—not significant.

It is well known that the studied compound, U-573, does not have cytotoxic activity against neuroblastoma cells. The EC_50_ value was found to be > 100 μM for U-573, as indicated in our previous investigation [28]. The cytoprotective activity of compound U-573 against the action of MPP+ (1 mM) for 24 h was studied on mouse neuroblastoma cells Neuro-2a. MPP+ itself reduced cell viability to 68.2%, while the use of U-573 increases it to 81.3%. BBG (10 μM) protected cell activity by 8.6% (Figure 2A).

The impact of U-573 on cell viability at elevated ATP concentrations (3 mM) was assessed using the MTT assay. To negate the cytotoxic effects of ATP (3 mM) on the cells, BBG (10 μM) was employed as a non-selective antagonist for the P2X7 receptor. The addition of ATP resulted in a 33.6% reduction in cell viability compared to the control group without ATP. Conversely, U-573 significantly enhanced cell viability in a dose-dependent manner in the presence of ATP, with increases of 7.7%, 12.1%, and 20.4% at concentrations of 0.1 μM, 1 μM, and 5 μM, respectively. Furthermore, treatment with BBG at a concentration of 10 μM led to a 16.3% improvement in cell viability (Figure 2B).

### 2.2. U-573 Inhibit ATP-Induced Ca^2+^ Influx in Neuro-2a Cells

**Figure 3:**
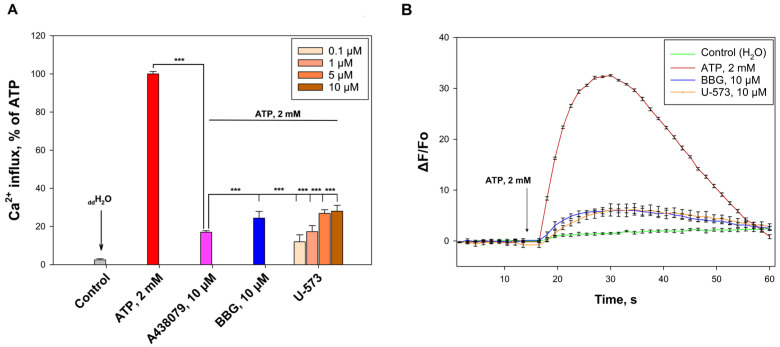
Compound U-573 inhibits ATP-induced Ca^2+^ influx in Neuro-2a cells: Effect of U-573 (0.1, 1, 5, and 10 µM), A438079 (10 µM), and BBG (10 µM) on Ca^2+^ influx, induced by ATP (2 mM) in Neuro-2a cells (**A**). Representative curves of [Ca^2+^]_i_ elevation induced by ATP alone or in the presence of U-573 (10 µM) or standard P2X7R blocker BBG (10 µM) (**B**). Data are presented as mean ± SE (*n* = 6). *** *p* < 0.001, compared to ATP.

P2X7 receptors (P2X7Rs) are non-selective ion channels that facilitate calcium ion permeability in response to extracellular ATP. Activation of these purinergic receptors results in an increase in intracellular calcium ion concentration ([Ca^2+^]_i_). To evaluate the ability of compound U-573 to inhibit P2X7Rs, neuroblastoma cells were loaded with the calcium-selective fluorescent probe Fluo-3AM. Upon adding ATP (2 mM) to Neuro-2a cells, a significant increase in [Ca^2+^]_i_ was detected. This calcium influx was effectively suppressed by the P2X7R standard blockers BBG (10 µM) and A438079 (10 µM). Studied compound U-573 at a concentration range of 0.1–10 μM demonstrated great inhibition of calcium influx, showing efficacy comparable to A438079 and BBG (Figure 3A,B). Compound U-573 showed inverse dose dependence and significantly reduced the influx of calcium ions at concentrations of 0.1, 1, 5, and 10 µM by 88.1%, 82.7%, 73.1%, and 71.9%, respectively. The treatment of cells with blockers A438079 (10 µM) and BBG (10 µM) led to the inhibition of ATP-induced calcium entry by 83.0% and 75.6%, respectively (Figure 3A,B).

### 2.3. U-573 Inhibit ATP-Induced EtBr and YO-TAP-1 Dyes Uptake in Neuro-2a Cells

The low-molecular-weight fluorescent dyes EtBr and YO-TAP-1 were selected to evaluate the capability of the studied compound U-573 to inhibit macropore formation, mediated by P2X7R in response to ATP action.

**Figure 4:**
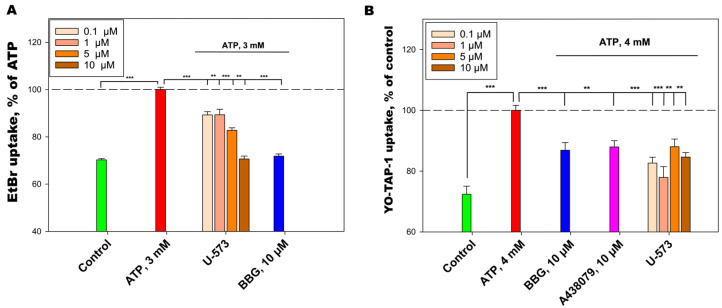
U-573 inhibits ATP-induced uptake of EtBr (**A**) and YO-TAP-1 (**B**) in Neuro-2a cells. Data are presented as mean ± SE (*n* = 6). ** *p* < 0.01, *** *p* < 0.001 compared to ATP.

The treatment of Neuro-2a cells by 3 mM ATP increases the EtBr uptake up to 42.3% relative to the control without ATP. In a dose-dependent manner, U-573 inhibited growth of EtBr fluorescence in cells at concentrations 0.1, 1, and 5 µM by 10.8%, 10.7%, and 17.3%, respectively. At a concentration of 10 µM, U-573 and BBG demonstrated comparable effects, with 29.4% and 28.2% of EtBr uptake inhibition, respectively (Figure 4A).

U-573 inhibited fluorescent dye YO-TAP-1 entry at concentrations of 0.1, 1, 5, and 10 µM by 17.4%, 22.1%, 12.0%, and 15.4%, respectively, without a dose-dependent effect. Standard P2X7R blockers A438079 and BBG at a concentration of 10 μM showed a comparable effect, inhibiting dye uptake by 12.1% and 13.1% (Figure 4B).

### 2.4. U-573 Inhibits MPP+-Induced NO and ROS Formation

The neuroprotective properties and antioxidant activity of U-573 were studied by exposing Neuro-2a mouse neuroblastoma cells to the neurotoxin MPP+ (1 mM).

**Figure 5:**
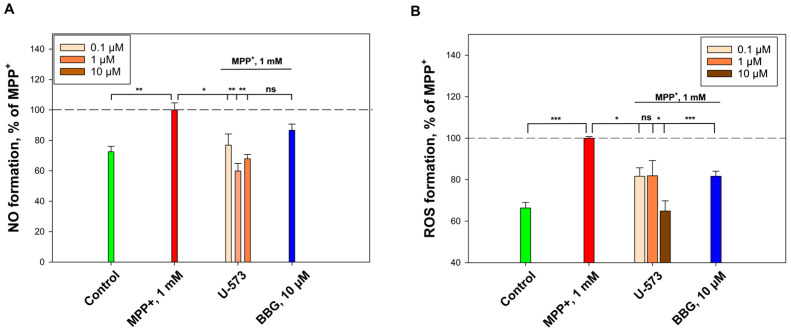
Anti-inflammatory effect of U-573 during the impact of MPP+ in Neuro-2a cells. Influence of U-573 on the intracellular formation of NO (**A**) and ROS (**B**). Data are presented as mean ± SE (*n* = 6). * *p* < 0.05, ** *p* < 0.01, *** *p* < 0.001 compared to MPP+; ns—not significant.

It was demonstrated that the addition of MPP+ (1 mM) increased the formation of nitric oxide by 27.5%. Compound U-573 significantly reduced NO production at concentrations of 0.1, 1, and 10 μM by 23.1%, 40.1%, and 32.1%, respectively. Meanwhile, BBG inhibited NO formation by 13.3% (Figure 5A).

Also, the effect of MPP+ (1 mM) increased the production of ROS by 33.6%, while the compound U-573 significantly inhibited the formation of reactive oxygen species in Neuro-2a cells at concentrations of 0.1, 1, and 10 μM by 18.3%, 18.1%, and 35.1%, respectively. BBG (10 μM) decreased ROS level by 18.4% (Figure 5B).

### 2.5. Anti-Inflammatory Activity of U-573 In Vivo

**Figure 6:**
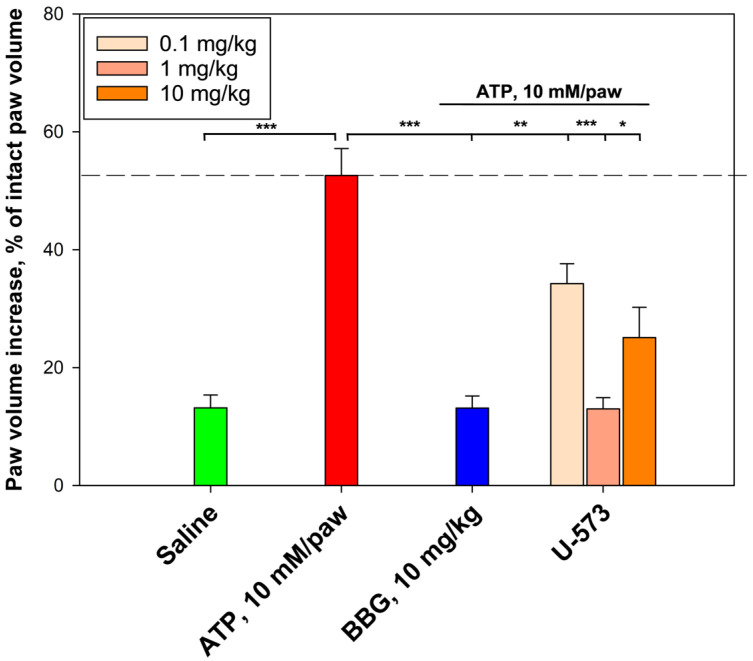
Anti-inflammatory effect of compound U-573 in a model of ATP-induced paw edema of mice. The dotted line indicates the level of mouse paw volume 1 h after ATP administration. Solutions of U-573 and BBG were administered intraperitoneally 60 min before the induction of inflammation. Paw volume was measured 1 h after intraplantar injection of ATP at a dosage of 10 mM/paw in a volume of 20 μL. The results obtained are presented as mean ± SEM (*n* = 6); * *p* < 0.05, ** *p* < 0.01, *** *p* < 0.001 compared to ATP.

The anti-inflammatory properties of U-573 were evaluated using a model of acute localized inflammation induced by ATP (Figure 6). Injection of ATP (10 mM per paw) into the pads of the mice’s hind legs resulted in a significant increase in paw edema after the administration of the inducer. This model is used to investigate the anti-inflammatory and antinociceptive effects of potential P2X7R inhibitors.

Injection of saline into the mouse paw caused only a slight increase in paw volume compared to the initial level of 13.2%. At the same time, induction of inflammation caused by ATP (10 mM/paw) resulted in increase in paw volume by 52.6%. A dosage of 1 mg/kg for the substance U-573 was the most effective and reduced edema by 39.6%, to the level of the control. The obtained result was identical to the effect of BBG at a dosage of 10 mg/kg, which also reduced edema size by 39.5%, relative to the increase caused by ATP. Compound U-573 showed a less inhibitory effect at other dosages (1 mg/kg and 10 mg/kg) (Figure 6).

### 2.6. U-573 Inhibits Protein Aggregation in Neuro-2a Cells Induced by MPP+

We studied the effect of compound U-573 on the aggregation of intracellular amyloid proteins during the induction of Parkinson’s disease in neuronal cells in vitro in the presence of the neurotoxin MPP+. Protein aggregation was quantitatively assessed using intravital cell staining with the fluorescent probe CRANAD-2, which primarily binds highly specifically to A*β*, followed by spectrofluorometry. Visualization of the forming protein aggregates was performed using fluorescence microscopy.

**Figure 7:**
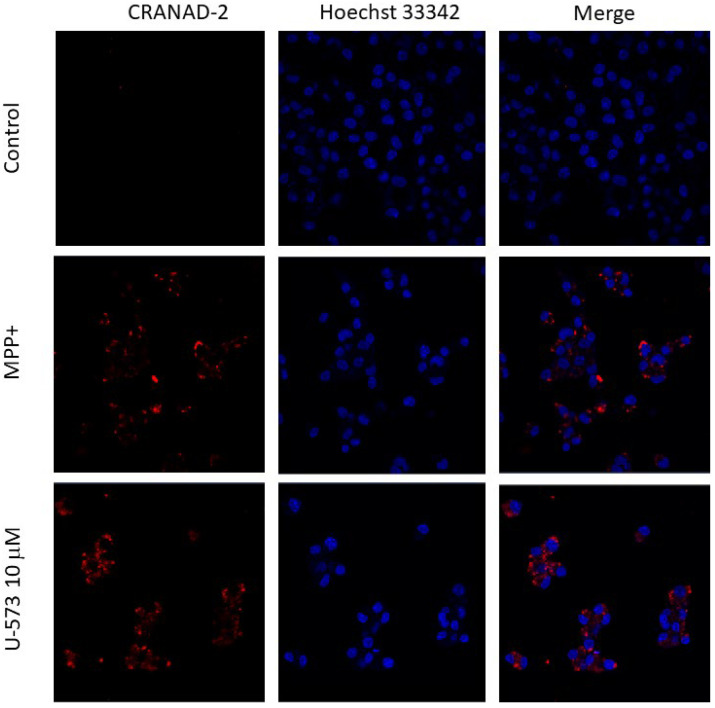
Protein aggregation in Neuro-2a cells in intact cell culture (Control), in the presence of MPP+ (1 mM), or MPP+ and U-573 (10 μM). Images of neuronal cells were obtained using a laser scanning confocal microscope LSM 710 LIVE AxioObserver at 40× magnification after cell staining with CRANAD-2. Cell nuclei are shown in blue (Hoechst 33342).

**Figure 8:**
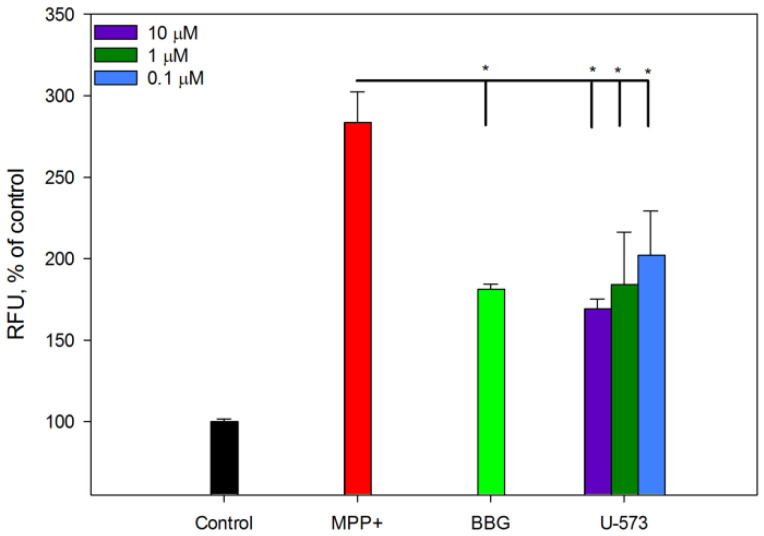
Spectrofluorimetric determination of U-573 influence on protein aggregation in Neuro-2a cells in the presence of MPP+ (1 mM). BBG (10 μM) was used as a standard P2X7R blocker. Data were obtained using a PHERAstar FS after cell staining with CRANAD-2. Data are presented as mean ± SE (*n* = 6). * *p* < 0.05 compared to MPP+ alone.

It was found that culturing neuronal cells in the presence of MPP+ leads to a sharp 2.5–3-fold increase in the fluorescence intensity of protein deposits detected in cells using the CRANAD-2 probe. In the cell images obtained using confocal microscopy, bright fluorescent aggregates filling the space of the cell cytoplasm are clearly visible (Figure 7 and Figure 8).

It was shown that the P2X7 receptor blocker BBG at a concentration of 10 μM significantly suppresses the fluorescence intensity of CRANAD-2-stained cells by approximately 50% (Figure 8), which is accompanied by a decrease in the number and brightness of cell fluorescence detected using fluorescence microscopy (Figure 7).

The studied 1,4-naphthoquinone U-573 also reliably, significantly, and dose-dependently suppressed the process of formation of protein aggregates in cells. This compound showed the greatest inhibitory activity at a concentration of 10 μM. Its effect was comparable to the effectiveness of BBG (Figure 7 and Figure 8).

### 2.7. ADME Analysis of U-573

To predict the probability of U-573 passing through the blood–brain barrier and to eliminate undesirable pharmacokinetics of this compound, the web-based platform ADMETlab 2.0 was used. This computer program allows evaluating 17 physicochemical properties, 13 medical chemistry measures, 23 ADME endpoints, 27 toxicity endpoints, and 8 toxicophore rules of the studied molecules.

**Figure 9:**
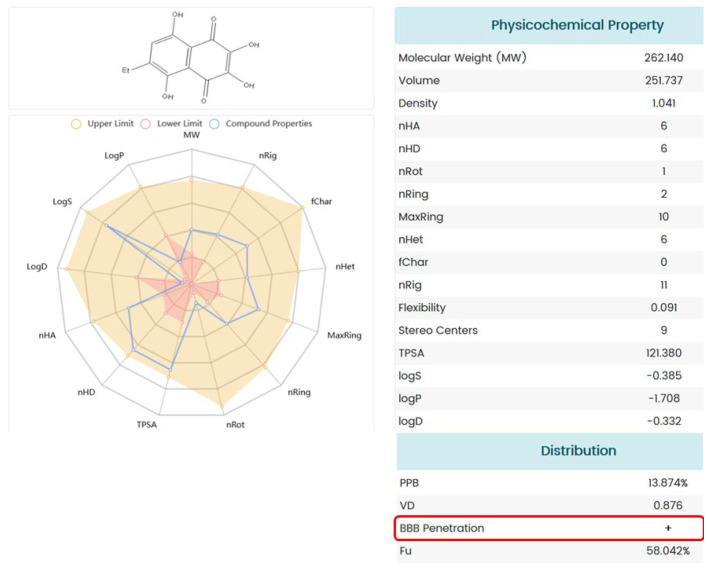
The ADME profile prediction of compound U-573 processed using ADMETlab 2.0. The red box shows the ability of U-573 to pass through the BBB.

The physicochemical properties of the studied molecule, discovered as a result of the analysis, allow us to assume with a high degree of probability that the low toxic compound U-573 is capable of passing through the BBB (Figure 9).

### 2.8. Evaluation of Cognitive Movement of Animals in a Mouse Model of Parkinson’s Disease

**Figure 10:**
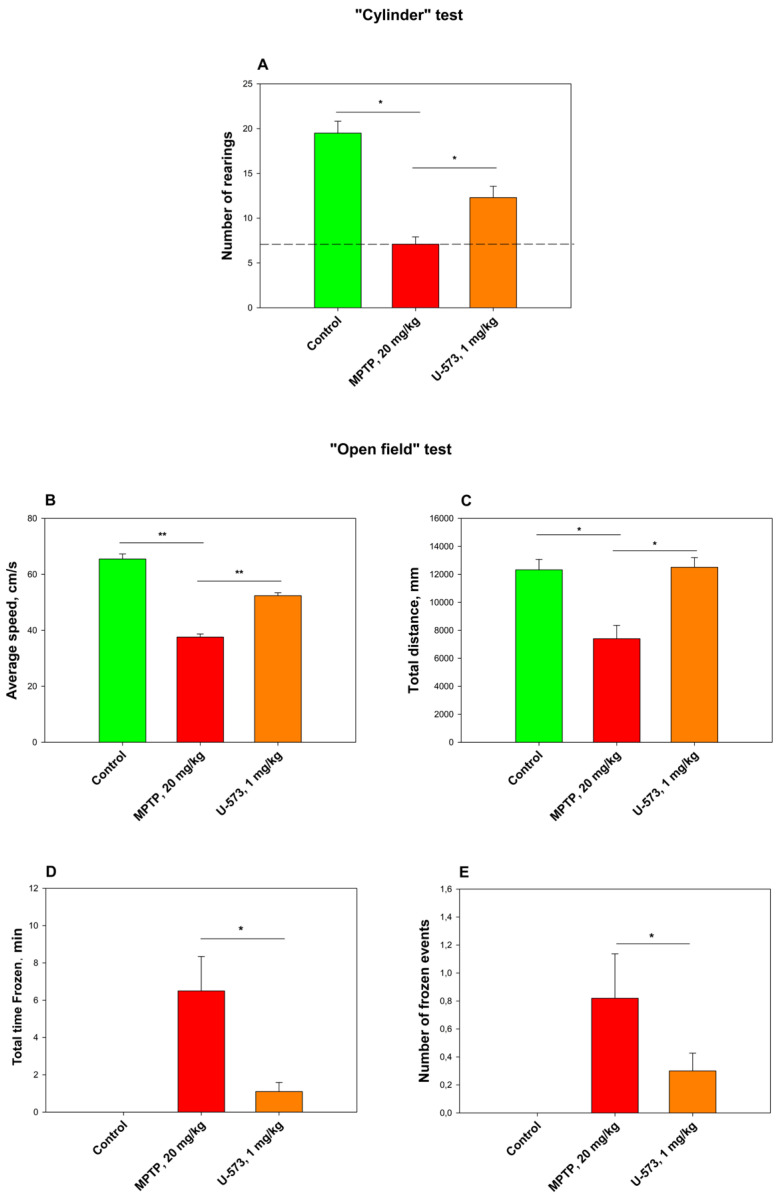
Effect of U-573 on spontaneous locomotion in MPTP-induced Parkinson’s disease mice. Effect of U-573 on the number of rearing of mice after MPTP administration in the “Cylinder” test (**A**), as well as on the average mouse speed (**B**), total distance traveled (**C**), total time in a frozen state (**D**), and the number of freezings in the “Open Field” test (**E**). U-573 was applied at a dosage of 1.0 mg/kg. Data are presented as mean ± SD (*n* = 6), * *p* < 0.05, ** *p* < 0.001 compared to the group of mice treated with MPTP alone.

The neurotoxin MPTP was used as an inducer of the Parkinson’s disease model. For this purpose, MPTP in physiological solution was administered to C57BL/6 mice at a dose of 20 mg/kg intraperitoneally once a day for 5 days. Compound U-573 at a dose of 1.0 mg/kg was administered intraperitoneally 24 h after the last MPTP injection 3 times every other day. The dose of 1 mg/kg was selected based on the previous in vivo experiment (Figure 6). Efficacy was assessed 24 h after the last administration of 1,4-naphthoquinone in the “Cylinder” and “Open Field” tests. The obtained results are presented in Figure 10.

From the data obtained, it can be seen that the neurotoxin MPTP significantly reduced the motor activity of the mice to 40–50% in the “Cylinder” test. The average speed of the mice and the total distance traveled increased significantly over time. During the “Open Field” test, the number of animal total time “frozen” decreased and the distance traveled increased.

A positive effect was observed when U-573 was administered at a dose of 1.0 mg/kg. This reflected in the improvement in the mouse cognitive behavior in the “Cylinder test”. At the same time, U-573 injections led to a significant and reliable increase in the mouse average movement speed and the distance traveled in the “Open Field” test, almost reaching the control values. The injection of U-573 significantly and reliably reduced the total time “frozen” by the mice in a stationary state.

## 3. Discussion

It is known that a number of natural 1,4-naphthoquinones and their synthetic derivatives have neuroprotective activity and are able to protect neuronal cells from toxic damage caused by neurotoxins such as paraquat (PQ) and 6-hydroxydopamine (6-OHDA). For example, among the library of 5,8-dihydroxy-1,4-naphthoquinone derivatives, consisting of 44 compounds, we recently discovered the five most active 1,4-naphthoquinone derivatives that prevent the toxic impact of these neurotoxins [28]. The two most active compounds from this group, acetylated 1,4-naphthoquinone-O-glucoside (U-443) and ethyl-2,3,5,8-tetrahydroxy-1,4-naphthoquinone (U-573), effectively protected Neuro-2a cells from the damaging effects of rotenone (Rot) [26,27].

One of the chemical compounds that reproduces the death of dopaminergic neurons in PD in animals is the substance MPTP (1-methyl-4-phenyl-1,2,3,6-tetrahydropyridine). Intracellular conversion of MPTP to MPP+ (1-methyl-4-phenylpyridine) most likely occurs in astrocytes under the action of the enzyme monoamine oxidase type B (MAO-B) [29]. The drug MPTP still remains a useful tool for creating animal models of Parkinson’s disease, while the use of MPP+ is the basis for creating models of neurotoxicity in cells, mainly on various lines of animal and human neuroblastoma cell cultures [30].

When penetrating neurons, MPP+ binds to the hydrophobic reaction site of NADH dehydrogenase, which leads to the cessation of oxidative phosphorylation, depletion of ATP, and cell death. In addition, the action of MPP+ causes the generation of ROS in dopaminergic neurons through a two-wave NADPH oxidase-dependent pathway [31]. In turn, excessive formation of ROS and reactive nitrogen species is a key event causing progressive neuronal damage in the development of a number of neurodegenerative diseases, including PD.

In current experiments, it was found that U-573 effectively inhibits NO and ROS formation induced by MPP+ in mouse neuroblastoma cells, which resulted in increased survival of neuronal cells in the presence of this neurotoxin. We have previously established that the cytoprotective effect of U-573 is associated with the ability to protect a number of intracellular enzymes from the inhibitory effects of different neurotoxins, protect biomembranes from lytic destruction, normalize the cell cycle and mitochondrial functions, suppress oxidative stress, reduce the levels of pro-inflammatory cytokines, exhibit antioxidant activity, and inhibit cyclooxygenase-2 (COX-2) activity in neuronal cells and macrophages [26]. In summary, it can be concluded that the antioxidant properties exhibited by this 1,4-naphthoquinone underlie the molecular mechanisms of its neuroprotective activity in the cellular model of MPP+ induced Parkinson’s disease.

In addition, the pronounced anti-inflammatory properties of ethylspinazarin were noted in in vivo experiments on an animal model in which inflammation was induced by injection of ATP into the paw. U-573 reliably and significantly reduced paw edema in mice and improved the general well-being of the animals. It is well known that ATP is a ligand for purinergic receptors of the P2X type, and activation of the P2X7 receptor subtype leads to powerful inflammation in neuronal cells and microglia [32,33]. In these experiments, the use of the P2X7 blocker BBG also led to almost complete elimination of the inflammation process induced in animals by ATP. This indicates that ATP-induced inflammation was mediated by activation of P2X7R.

We have shown that U-573 effectively blocks the functioning of P2X7 receptors in neuronal cells. This is primarily expressed in the blocking of Ca^2+^ influx into neuroblastoma cells under the influence of a high concentration of ATP, as well as in the inhibition of the ATP-stimulated entry of fluorescent dyes, EtBr, and YO-TAP-1, which generally indicates inhibition of ATP-induced formation of the P2X7 receptor macropore. This blocking effect is comparable to the action of known antagonists of this purinergic receptor, A438079 and BBG. Thus, the cytoprotective and anti-inflammatory properties of the studied ethylspinazarin may be associated not only with its antioxidant properties, but also with its ability to directly block P2X7 receptors.

It is known that one of the signs of PD in neurons is the aggregation of amyloid proteins, primarily *α*-Syn, which is considered one of the markers of this disease. The presence of toxic oligomers of this protein leads to disruption of the functioning of neurons, including mitochondrial dysfunction, endoplasmic reticulum stress, proteostasis dysregulation, synaptic impairment, cell apoptosis, neuroinflammation, and finally their death [34]. It is known that MPP+ and some other neurotoxins promote *α*-Syn oligomerization in neuronal cells [35]. In the present study, we showed that MPP+ also causes aggregation of amyloid proteins in neuronal cells. In our experiments, to detect intracellular protein aggregates, we used the fluorescent probe CRANAD-2, which binds with high selectivity primarily to A*β*, a marker of Alzheimer’s disease [36]. There is no contradiction in this, since evidence of coaggregation of at least three key amyloid proteins, *α*-Syn, A*β*, and tau-protein, in the induction and occurrence of neurodegenerative diseases such as Parkinson’s and Alzheimer’s diseases and some others has now been clearly established. Thus, in Lewy bodies, the histopathological hallmark of PD, not only was the sole aggregation of *α*-Syn reported but so was the joint aggregation of A*β* and tau, suggesting a protein triumvirate under these pathological conditions. Moreover, it is now known that the oligomerization of one amyloid protein can serve as a trigger for the onset of fibril formation of another, as has been established for A*β* and *α*-Syn recently [37,38]. Inhibition of the process of MPP+-induced, amyloid-like protein aggregation in neuroblastoma cells in the presence of naphthoquinone U-573 once again emphasizes its pronounced neuroprotective properties. The fact that BBG also effectively inhibits protein aggregation indicates the participation of purinergic receptors P2X7 in the aggregation process.

The tested compound U-573 showed pronounced efficacy as an antiparkinsonian agent in the “Cylinder” and “Open Field” tests with experimental animals. It is very likely that at the early stage of PD development caused by MPP+ intoxication in living organisms, the studied compound U-573 exhibits antiparkinsonian properties due to its ability to reduce the content of reactive oxygen species and nitric oxide in neurons. Since MPP+/MPTP can modulate the P2X7 receptor and upregulate the expression of P2X7Rs, as demonstrated in studies involving in vitro PC12 neuronal cells causing the process of neuroinflammation [39], one of the probable mechanisms of neuroprotection from the damaging effect of the neurotoxin MPP+ in the PD model in vivo may not only be the antioxidant activity of U-573, but also its ability to block the functioning of P2X7Rs, the overactivation and/or overexpression of which leads to the development of this disease. Since we did not find in previous studies a dose dependence in the antiparkinsonian effect of U-573 in the dose range of 0.1–10.0 mg/kg in the rotenone-induced Parkinson’s disease model in mice [26,27], in the present experiment, we used only a single dose of 1 mg/kg naphthoquinone, which was quite effective.

The ADME analysis revealed the potential for the 1,4-naphthoquinone under study to cross the BBB. This means that, theoretically, compound U-573 is capable of penetrating the brain of animals with PD and directly affecting inflammatory processes and molecular targets of neuroinflammation, including P2X7 receptors, thereby exerting a neuroprotective effect.

We have previously noted that a number of 1,4-naphthoquinones, including ethylspinasarin (U-573), are capable of protecting neuronal and macrophage cells from the toxic effects of PD inducers such as Rot, PQ, and 6-OHDA, reducing levels of pro-inflammatory cytokines TNF and IL-1β, notably inhibiting the activity of cyclooxygenase-2 (COX-2), and restoring mitochondrial membrane potential [26,28]. In addition, a number of synthetic compounds from this group have been shown to be able to effectively block the functioning of P2X7 receptors, which leads to a sharp reduction in pain and inflammation [21,22,23,24,25].

In the present study, we have shown for the first time that U-573 has the potential to inhibit P2X7 receptors in neuronal cells, and this property, along with others, may also be of key importance in the manifestation of the antiparkinsonian activity of this compound.

This work has a number of certain limitations due to the incomplete set of experimental approaches used in this area of research. Subsequently, our future exploration aims to extensively investigate the off-target effects of ethylspinasarin, which may also play a role in the antiparkinsonian activity of this compound with the application of P2X7R knockout and additional selective antagonists of this receptor. Moreover, we intend to evaluate the number of dopaminergic neurons in the substantia nigra in mice with induced Parkinson’s disease and the concentration of dopamine, to assess the degree of α-synuclein and tau-protein aggregation in the brain of mice, as well as to assess the pharmacokineics of U-573 in the body of experimental animals.

## 4. Materials and Methods

### 4.1. U-573 Compound

Ethylspinazarin (U-573) was prepared by chemical synthesis according to the procedures described previously [38,39] and was identical to natural pigment, firstly isolated from the sea urchin *Scaphechinus mirabilis* [37]. Solution of U-573 was prepared as a 10 mM stock solution in DMSO, which was adjusted with ddH2O to the final concentrations immediately before the experiments.

### 4.2. Cells Culture

Mouse neuroblastoma Neuro-2a cells (American Type Culture Collection (ATCC)^®^ CCL-131™) were procured from ATCC (Manassas, VA, USA). The cells were cultured in a 75 cm^3^ culture flask (Corning, Phoenix, AZ, USA) within a CO_2_ incubator using Dulbecco’s Modified Eagle’s Medium (DMEM) supplemented with 10% fetal bovine serum and 1% penicillin/streptomycin antibiotics (Biolot, St. Petersburg, Russia).

### 4.3. Cell Viability Estimation

Neuro-2a cells were seeded in DMEM in 96-well plates (2 × 10^4^/well), and then incubated for 24 h. Then, the studied compound was added to the medium in a volume of 20 μL (ddH_2_O in controls). The cells were incubated with the tested compound for 1 h, then ATP (3 mM) or MPP+ (1 mM) were added to the medium, after which the cells were incubated for another 24 h. After incubation, the cell medium was completely removed and 100 μL of fresh DMEM and 10 μL of MTT ((3-(4,5- dimethylthiazol-2-yl)-2,5-diphenyltetrazolium bromide) (Sigma-Aldrich, Burlington, MA, USA)) solution were added to the plate. The cells were then incubated for 4 h. After that, another 100 μL of SDS/HCL were added to the cells and the plates were left overnight. The next day, dye absorption was measured at a wavelength of 570 nm using a PHERAstar FS plate reader (BMG LABTECH, Ortenberg, Germany). Cell viability was estimated as percent of control data.

### 4.4. Calcium Uptake

Neuro-2a cells were plated in 96-well plates at a density of 2 × 10^4^ cells per well in DMEM and incubated overnight at 37 °C in a 5% CO_2_ atmosphere. The cells were then washed once with HBSS saline (pH 7.4) and subsequently loaded with 5 µM Fluo-3 AM (Abcam, Cambridge, UK) and 0.05% (*w*/*v*) Pluronic^®^ F-127 (Sigma-Aldrich, Burlington, MA, USA) in the same buffer solution. After loading, the cells were incubated for 40 min at 37 °C in a 5% CO_2_ environment, washed with HBSS saline without the fluorescent dye, and treated with the test compounds for 20 min at room temperature in the dark. Standard inhibitors, including the non-selective P2X7 receptor antagonist BBG (Brilliant Blue G, Sigma-Aldrich, Burlington, USA, final concentration 10 µM) and the competitive P2X7 receptor antagonist A438079 (10 µM; Sigma, Burlington, MA, USA), were employed as inhibitory controls. Fluorescence intensity was recorded using a PHERAstar FS plate reader (BMG LABTECH, Ortenberg, Germany) by measuring excitation at 490 nm and emission at 510 nm. ATP (HiMedia Laboratories, Thane, India, final concentration 2 mM) was added via a robotic microinjector following baseline recording.

### 4.5. EtBr Uptake

Neuro-2a cells were seeded in DMEM in 96-well plates (2 × 10^4^ cells/well), and then incubated for 24 h. Then the medium was replaced with 160 μL HBSS saline (140 mM NaCl, 5 mM KCl, 0.8 mM MgCl_2_, 2 mM CaCl_2_, 10 mM glucose, 10 mM HEPES, pH 7.4) and the tested substance were added at 20 μL to the cells for 30 min. Then 20 μL EtBr (ethidium bromide, Sigma, CA, USA) at final concentration of 5 μM were added to the cells and after 15 min of incubation, ATP solution (final concentration 3 mM) was added for 10 min. Then the cells were washed with HBSS solution without dye. Fluorescence was measured using a PHERAstar FS plate reader (BMG LABTECH, Ortenberg, Germany) at wavelengths λ_ex_ = 540 nm and λ_em_ = 590 nm. The results were presented as percentage of ATP data (positive control).

### 4.6. YO-TAP-1 Uptake

Neuro-2a cells (2 × 10^4^ cells/well) were seeded in DMEM in 96-well plates and incubated for 24 h. The medium was then replaced with HBSS saline containing the fluorescent dye YO-TAP-1 (Lumiprobe RUS, Moscow, Russia) at a concentration of 2.5 μM for 15 min. The studied compound and standard P2X7R antagonist BBG (10 μM) were then added to the cells, resulting in an incubation of the cells for 10 min. After that, ATP (4 mM) was added to the cells for an additional 10 min. After incubation with the inducer, the cells were washed twice with HBSS saline without dye. Dye fluorescence was measured using a PHERAstar FS plate reader (BMG LABTECH, Ortenberg, Germany) at λ_ex_ = 485 nm and λ_em_ = 520 nm in the “End Point” mode. The effectiveness of compounds was evaluated relative to control without ATP.

### 4.7. Analysis of ROS and NO Levels

Neuro-2a cells (2 × 10^4^ cells/well) were treated with compound U-573 at concentrations ranging from 0.01 to 10 μM for a duration of 1 h. Following this treatment, MPP+ (Sigma-Aldrich, Burlington, USA) was added to each well to achieve a final concentration of 1 μM, and the cells were incubated for an additional hour. To assess the generation of reactive oxygen species (ROS), a 20 μL aliquot of 2,7-dichlorodihydrofluorescein diacetate (H2DCF-DA, Molecular Probes, Eugene, USA) was added to each well at a final concentration of 10 μM. The plates were incubated at 37 °C under conditions of complete darkness for 10 min. For the measurement of nitric oxide (NO) levels, the NO-sensitive probe DAF-FM DA (Molecular Probes, Eugene, USA) was introduced into each well at a concentration of 5 μM. The plates were incubated at 37 °C under conditions of complete darkness for 40 min. Fluorescence intensity was quantified using a PHERAstar FS high-speed reader (BMG Labtech, Ortenberg, Germany), with excitation set at 485 nm and emission detected at 520 nm. The resulting data were analyzed using MARS Data Analysis software version 3.01R2 (BMG Labtech, Ortenberg, Germany), and the results were expressed as a percentage of the control.

### 4.8. Amyloid-β Aggregation

Neuro-2a cells (2 × 10^4^ cells/well) were exposed to compound U-573 at concentrations varying from 0.01 to 10 μM for a duration of 1 h. To inhibit the P2X7 receptor, BBG was added to each well at a final concentration of 10 μM. Following this, MPP+ was introduced to each well at a final concentration of 1 μM, and the cells were incubated for an additional 24 h. To evaluate amyloid-*β* (A*β*) aggregation in the neuronal cells, a 20 μL solution of the fluorescent probe CRANAD-2, prepared in 10% DMSO, was added to each well at a final concentration of 50 μM. The plates were subsequently incubated in the dark at 37 °C for 30 min. Following the incubation period, the cells were washed twice with PBS (pH 7.4), and the fluorescence intensity was assessed using a PHERAstar FS high-speed plate reader (BMG Labtech, Ortenberg, Germany), with excitation set at 540 nm and emission at 590 nm. The data obtained were analyzed using MARS Data Analysis software version 3.01R2 (BMG Labtech, Ortenberg, Germany), and results were expressed as a percentage of the control. The synthesis of CRANAD-2 followed the methodology outlined in the literature [40].

For the visualization of intracellular A*β* aggregates, Neuro-2a cells were seeded in a 6-well plate at a density of 5 × 10^4^ cells/mL and allowed to adhere for 24 h. A cover glass measuring 30 × 0.17 mm (Paul Marienfeld GmbH & Co. KG, Germany) was placed in each well prior to cell plating. The cells were treated with compound U-573 and other reagents as previously described. A*β* aggregation in the neuronal cells was assessed after staining with CRANAD-2 using a laser scanning confocal microscope LSM 710 LIVE AxioObserver (Carl Zeiss GmbH, Jena, Germany). The fluorescence of CRANAD-2 was excited using a 543 nm laser, and the emission was recorded in the range of 609 nm fluorescence of Hoechst 33342 was excited at 405 nm, with emission detected at 459 nm. Image processing and subsequent analysis were performed using ZEN 2011 software (Carl Zeiss GmbH, Jena, Germany).

### 4.9. Animals

Female mice of C57BL/6 strain (weighing 20–22 g, 2-month age), were obtained from the Russian National Center for Genetic Resources of Laboratory Animals. The mice were divided into experimental groups of six animals each and housed in a vivarium condition under a controlled 12 h light/dark cycle at a temperature of 22 ± 1 °C, with unrestricted access to food and water. All experimental procedures were conducted in accordance with the International Recommendations for Biomedical Research Using Animals, endorsed by the International Council of Medical Scientific Societies (CIOMS), the Order of the Ministry of Health and Social Development of Russia dated 23 August 2010, No. 708n, “On Approval of Laboratory Practice Rules” and approved by the local ethics committee of the G.B. Elyakov Pacific Institute of Bioorganic Chemistry, Far Eastern Branch of the Russian Academy of Sciences (protocol No 01/20 of 9 September 2020).

### 4.10. ADMET Analysis

The ADMET profile of U-573 was estimated using ADMETlab 2.0 [40]. The molecular SIMLE strings were generated using Chemdraw and then submitted to the online system. An analysis report was then generated.

### 4.11. Paw Edema Assay

A model of acute inflammation of the swelling of a mouse paw to study the anti-inflammatory effect of U-573 was used. The inflammation was induced by subplantar injection of ATP solution into the left hind paw of female C57BL/6 mice. Each mouse was individually marked. The mice were divided into groups of 6 individuals. The groups were as follows: 1—intact; 2—control solvent DMSO; 3—control ATP; 4, 5, 6—experimental groups with different doses of U-573; 7—BBG control. The initial volume of the paw was measured using a plethysmometer (Ugo basile 37140, Milan, Italy), which was taken as 100%. U-573 was injected intraperitoneally at doses of 0.1, 1, and 10 mg/kg, 60 min before the treatment of ATP. The testing U-573 was diluted in sterile water from the stock solution in DMSO (10 mM) at different dosages. The final concentration of DMSO was 1%. ATP solution was diluted in physiological solution and was injected into the left hind paw of the mouse in dosage of 10 mM in volume of 20 μL. Standard antagonist of P2X7R BBG at a dose of 2.5 mg/kg was injected intraperitoneally. Control solvents were injected according to the scheme with experimental groups in mice. After 1, 2, 4, 6, and 24 h, the paw edema was measured using a plethysmometer. The increase in paw volume after the injection was expressed as a percentage of the healthy intact paw volume.

### 4.12. Parkinson’s Disease Induction

Parkinson’s disease was induced in mice according to [41,42] with some modifications. After acclimatization, the mice were randomly divided into four groups: the first group consisted of control mice in their normal state; the second group received a control solvent; the third group received MPTP (1-methyl-4-(2′methyl-phenyl)-1,2,3,6-tetrahydropyridine hydrochloride, Sigma-Aldrich, Burlington, MA, USA) at a dose of 20 mg/kg per day for five days, subcutaneously, in the withers area (negative control); and the fourth group received U-573 (1 mg/kg) and MPTP compounds intraperitoneally. The test compound U-573 was dissolved in DMSO (the final concentration is 1%) and administered intraperitoneally at a dose of 1 mg/kg, three times every other day. The mice behavior was assessed the day after the last injection using the “Cylinder” and “Open Field” tests.

### 4.13. “Cylinder” Test

The transparent glass cylinder, with a height of 19.5 cm and a diameter of 15.0 cm, was partially shielded on three sides with black cardboard to reduce the effects of ambient light. The side facing the camera was left unobstructed for video recording. A SONY digital camera (Carl Zeiss, Vario-Tessar, 70×) was utilized for the experiment, capturing mouse behavior over a duration of 3 min. To ensure that the mouse behavior was not disturbed, precautions were taken to minimize noise and light fluctuations during the recording. Following the session, the cylinder was thoroughly washed with water, and the inner surface was sanitized using 70% ethanol to remove any residual mouse odors and then allowed to dry before introducing a new mouse. Changes in the mouse behavioral responses were evaluated in relation to the development of the PD MPTP-model, and the total number getting up on hind legs.

### 4.14. “Open Field” Test

For the Open Field test, an opaque plastic box measuring 100 × 100 × 70 cm was utilized. Before the assessment, all mice were given a 5 min preadaptation period inside the box. Following this, a pair of mice was gently positioned in the center of the open field, and their movements were recorded for 3 min using a SONY digital camera (Carl Zeiss, Vario-Tessar, 70×). The software “ToxTrac” version 2.84 (Umea University, Linneavag, SE-90187, Sweden) was used to assess animal behavior, collect data, and perform analysis. Measurements were made of average speed (cm/min), total distance traveled (mm), total time “frozen” (spent in stop, min), and number of stop cycles. Between tests, the container was thoroughly washed with a solution of 10% ethanol and water.

### 4.15. Statistics

Statistical analysis was conducted using SigmaPlot 14.0 software (Systat Software Inc., San Jose, CA, USA). For multiple comparisons, one-way analysis of variance (ANOVA) followed by a Tukey HSD test were applied. Results are expressed as means ± standard error of the mean (SEM), with significance defined as *p* < 0.05.

## 5. Conclusions

The aim of this work was to study the ability of the pigment of the sea urchin 1,4-naphthoquinone ethylspinazarine (U-573) to protect mouse Neuro-2a neuronal cells from the neurotoxic effect of one of the Parkinson’s disease inducers, MPP+. We established the aptitude of this compound to block Ca^2+^ influx and inhibit macropore formation through the P2X7 receptor induced by high concentrations of ATP. The effectiveness of compound U-573 in reducing neuroinflammation, as well as ROS and NO formation in neuronal cells under the influence of ATP and MPP+, and preventing MPP+-induced formation of amyloid-like protein aggregates in neuronal cells was demonstrated. In addition, the potential of ethylspinazarin in providing anti-inflammatory and antiparkinsonian effects was demonstrated using a mouse model of ATP-induced inflammation and MPTP-induced Parkinson’s disease. It is likely that the antiparkinsonian activity in in vivo experiments may be mediated by the capability of U-573 to cross the BBB. Finally, we found that U-573 effectively inhibits the functioning of ATP-dependent purinergic P2X7 receptors in neuronal cells. This property, among others, may be of key importance in the manifestation of the antiparkinsonian activity of this 1,4-naphthoquinone.

## Data Availability

The data presented in this study are available upon request from the corresponding author.

## Abbreviations

The following abbreviations are used in this manuscript:

P2X7R	P2X7 receptor
ATP	Adenosine triphosphate
PD	Parkinson disease
α-Syn	Alpha-synuclein
BBB	Blood–brain barrier
A*β*	Amyloid-*β*
TNF-α	Tumor necrosis factor α
IL-1β	Interleukin 1β
IL-18	Interleukin 18
ROS	Reactive oxygen species
NO	Nitric oxide
BBG	Brilliant blue G
COX-2	Cyclooxygenase-2
